# Hazardous Chemical Compounds in Cookies: The Role of Sugars and the Kinetics of Their Formation during Baking

**DOI:** 10.3390/foods11244066

**Published:** 2022-12-16

**Authors:** Biagio Fallico, Antonia Grasso, Elena Arena

**Affiliations:** Dipartimento di Agricoltura, Alimentazione e Ambiente (Di3A), University of Catania, Via Santa Sofia 98, 95123 Catania, Italy

**Keywords:** α-dicarbonyl compounds, 3-deoxyglucosone, glyoxal, methylglyoxal, 5-hydroxymethylfurfural, activation energy

## Abstract

Baking goods are an essential part of the diet worldwide and are consumed daily, so they represent ideal foods for vehicle health- and unhealth-promoting substances. This work aimed to study the influence of sugars and baking conditions of cookies on the final levels of the main reported hazardous chemical compounds such as 5-hydroxymethylfurfural (HMF), 3-deoxyglucosone (3-DG), glyoxal (GO) and methylglyoxal (MGO). The replacement of sucrose with fructose or glucose in the cookies recipe deeply modifies the levels of α-dicarbonyl compounds (DCs), particularly 3-DG, independently of the baking temperature used. A longer baking time, even a few minutes, can drastically modify the HMF level in cookies and the use of fructose or glucose in the recipe seems to ensure the optimal conditions for generating this compound. The use of sucrose is required to keep levels of the hazardous compounds below a few mg/kg. Additionally, the ability to retain water, the titratable acidity and/or the pH of the final products were influenced by the used sugars with effects on the final levels of DCs and HMF. The highest Ea values determined for DCs and HMF formation in the cookies with sucrose suggest that this system requires very high temperatures to increase meaningful levels of these molecules, limiting their accumulation.

## 1. Introduction

Baking goods are an indispensable part of the diet all over the world and are consumed daily; therefore, they represent ideal foods both as vehicle health- and unhealth-promoting substances.

In recent years, numerous studies on baked goods have been undertaken due to safety concerns about hazardous chemical products formed during processing, particularly α-dicarbonyl compounds (DCs), furan, 5-hydroxymethylfurfural (HMF), furosine and acrylamide [1,2,3,4].

These compounds are reactive Maillard reaction products that can react with other compounds, leading to the desired formation of compounds crucial for flavor, aroma and color development [5,6,7]. In addition to the positive effect on baked goods of these compounds, several adverse effects on health were reported. DCs are related to the etiology of several chronic diseases, such as type 2 diabetes and diabetic-related complications [4,5,6,8,9,10,11], rheumatoid arthritis [12], and chronic kidney diseases [13,14]. DCs readily react with proteins or α-amino groups of amino acids to form the advanced glycation end products (AGEs) associated with several chronic degenerative diseases such as cataracts, Alzheimer’s disease, cardiovascular diseases, and atherosclerosis [15,16,17]. Moreover, cytotoxic activity, cancer-promoting effects, and the detrimental effect on the gut microbiota of DCs were reported [18,19,20]. Recently, it was highlighted that the postprandial levels of DCs depend on many variables, including physiological state, amount, and composition of food, although food intake of 3-DG was not significantly correlated with plasmatic levels of 3-DG or skin autofluorescence [21]. In terms of HMF, it can be converted in vitro and in vivo into 5-sulfoxymethylfurfural, which is known to induce genotoxic and mutagenic effect [22,23].

It is well known that the chemical mechanism of the Maillard reaction is complex and difficult to elucidate [24]. DCs are precursors of advanced glycation end products (AGEs) and other intermediates [25] and are also difficult to predict kinetically, both in general systems [26,27] and in model systems of baked goods [28].

Research has been conducted on the quality of sweet baked goods as a function of baking temperature and two critical periods during baking have been identified: the heating step, where the temperature rises linearly before plateauing, and the final phase, which corresponds to dehydration of the crust while the inner temperature remains at approximately 100 °C [29].

The effects of cookie formulation and baking conditions [30], as well as the role of NaCl and temperature–time profiles on acrylamide and HMF levels, were investigated [31]. The role played by the type of sugar [32,33,34,35,36], fats, and leavening agent on DCs, acrylamide and HMF production in cookies has also been investigated [33,37,38]. The use of a sugar alcohol, such as maltitol instead of sugar, strongly limited HMF production in cookies [39]. Moreover, the high variability of DCs in commercial cookies has been reported due to different cookie processing and recipes [40,41].

The aim of this paper was to determine the role of different sugars (sucrose, glucose and fructose) and baking conditions on the development of the three most common DCs —3-DG, GO and MGO and HMF—in cookies. Moreover, these data were used, for the first time, for kinetics studies on cookies and to suggest a reduction in and/or control strategy for limiting the formation of these hazardous chemicals compounds during their production.

## 2. Materials and Methods

### 2.1. Chemicals

Sucrose, fructose, glucose, o-phenylenediamine (OPD) and 5-hydroxymethyl furfural (HMF) with high purity (>98%), and glyoxal (GO; 40% in water) and methylglyoxal (MGO; 40% in water) were purchased from Sigma-Aldrich (St. Louis, MO, USA). 3-deoxyglucosone (3-DG; purity 95%) was purchased from Santa Cruz Biotechnology, Inc. (Santa Cruz, CA, USA). All the solvents for HPLC analysis were from JT Baker (Deventer, Holland).

### 2.2. Cookies Preparation

Cookies were prepared using the recipe described in the method 10–54 [42] with slight modifications. The recipe for sucrose cookies was as follows: 80 g of wheat flour, 33.6 g of sucrose, 32 g of butter, 1 g of salt, 0.8 g of sodium bicarbonate, 0.4 g of ammonium bicarbonate and 17.6 mL of deionized water. The modified recipe had the same value for total solvent, %S and % fat of the original recipe (Kweon et al., 2010). The sucrose was replaced with fructose (fructose cookies) or glucose (glucose cookies) and both dough mixing and baking were conducted in duplicate. The dough was kneaded using a dough mixer (model 1596, Ariete, Italy), laminated to a uniform thickness (2 mm) using a pasta machine (Imperia, Lusso, sp 150 model, Bologna, Italy), and formed into discs with a stainless-steel cutter with a diameter of 5 cm. The cookies were baked in a laboratory oven (ThermoScientific, Waltham, MA, USA) at three different temperatures of 150, 170 and 190 °C and various baking times (5, 10, 15, 20, 25, 30, and 35 min at 150 °C; 5, 10, 15, 20, and 25 min at 170 and 190 °C). Baking temperatures and time used in this study were set based on cookie thickness and color development and were in the range suggested for short doughs [43]. After baking, cookies were immediately cooled and ground in a home grinder (La Moulinette, Moulinex, Écully, France) before chemical analyses.

### 2.3. Chemical Characterization

Moisture content was determined by oven-drying gravimetric analysis using an oven (ThermoScientific, Herathermoven, Italy) at 105 °C for 4 h. pH values and the total titratable acidity (TTA) were measured with a pH meter (Mettler Toledo, MP 220, Milano, Italy). An amount of 10 g of grounded sample was mixed with 90 mL of distilled water and the suspension was titrated with 0.1 M NaOH to a pH of 8.5. TTA was expressed on dry matter as mL of NaOH needed for titration [44]. The reported result of each analytical determination is the average of six values (2 sample replicates × 3 analytical replicates).

For α-dicarbonyl compounds (DCs) and HMF extraction, an aliquot of the milled sample (1 g) was put into a volumetric flask (10 mL) and 5 mL of deionized water was added. After 10 min of stirring, the solution was brought to volume of 10 mL with deionized water and centrifuged at 10 °C for 15 min at 8500 rpm (ALC 4128, Italy). An aliquot of the supernatant was filtered through a 0.45 μm filter (Albet) and directly analyzed to determine HMF content, while DCs were derivatized prior HPLC analysis.

### 2.4. HPLC Analysis

#### 2.4.1. α-Dicarbonyl Compounds (3-DG, GO, and MGO)

A volume of 1 mL of the filtered supernatant was derivatized with a 0.6% OPD solution in water; and after 12 h in the dark, the derivatized mixture was injected into an HPLC (Spectra System) equipped with a diode array detector (UV6000LP) and an autosampler (AS3000) (Thermo Electron, San Jose, CA, USA). The chromatographic separations were performed on a Luna C18 (250 mm × 4.6 mm, 5 μm) (Phenomenex, Torrance, CA, USA) using a gradient mixture of (A) 0.1% (*v/v*) acetic acid in water and (B) methanol as the mobile phase, at a flow rate of 0.7 mL/min; the injection volume was 20 μL. The gradient program was: t0 85% A and 15% B; t10 65% A and 35% B; t15 35% A and 65% B; t25 100% B; t30 85% A and 15% B. The detector wavelength was set to 312 nm [45]. All compounds were identified by comparing retention times and UV spectra to those of standard solutions and by spiking each sample with standards. Quantification of each DC was performed using external calibration curves. The extraction procedure and the analyses were performed in triplicate, the reported concentration of each dicarbonyl compound was therefore the average of six values (2 sample replicates × 3 analytical replicates). The results were expressed as mg of dicarbonyl compound/kg of cookies dry matter.

#### 2.4.2. HMF

An aliquot of the filtered supernatant was injected into an HPLC system (Shimadzu Class VP LC-10aDvp) equipped with a DAD (Shimadzu SPD-M10Avp). A Gemini NX C18 (150 mm × 4.6 mm, 5 μm) (Phenomenex, Torrance, CA, USA) column fitted with a guard cartridge packed with the same stationary phase was used. The HPLC conditions were as follows: 0.1% (*v/v*) acetic acid in water (94%) and methanol (6%); flow rate, 0.7 mL/min; injection volume, 20 μL; and chromatograms were monitored at 283 nm [46].

HMF was identified by comparing retention times and UV spectra from samples with a standard solution and by splitting each sample with the HMF standard. Quantification of HMF was performed using external calibration curves. All analyses were performed in triplicate, including the extraction procedure, and the reported HMF concentration was therefore the average of six values (2 sample replicates × 3 analytical replicates). The results were expressed as mg of HMF/kg of cookies dry matter.

### 2.5. Kinetics and Statistical Analysis

All results were expressed as the mean value ± standard deviation (SD).

The rates of 3-DG and HMF formation were obtained through the following equation:

[A]_t_ − [A]_0_ = kt (1)

where [A] is the concentration of 3-DG, GO, MGO or HMF, k is the kinetics constant (min^−1^) extrapolated by plotting [A]_t_ to [A]_0_ compared with time (t), and t is the heating time (min). The activation energy Ea (kJ/mol) values of 3-DG, GO, MGO and HMF formation were calculated from the rate coefficients at different temperatures by applying the Arrhenius equation:

(2) $$\ln K=\mathrm{lnA}-\frac{\mathrm{Ea}}{\mathrm{RT}}$$

where k is the kinetics constant (min^−1^), A is the pre-exponential factor (min^−1^), Ea is the activation energy (J/mol), R is the universal gas constant (8.314 J/mol K), and T is the temperature (K).

For each parameter (moisture, pH, TTA, HMF, 3-DG, GO, MGO), experimental data determined on cookie samples baked at the same temperature and time but containing different sugars (sucrose, fructose and glucose) were subjected to analysis of variance (ANOVA) with the significance defined at (*p* < 0.05). Significant differences (mean separation) between these cookie samples were determined by Tukey’s test (*p* < 0.05). All statistical evaluations were conducted using the Minitab 19 (USA) statistical analysis software.

## 3. Results and Discussion

### 3.1. Evolution of Chemical Parameters of Cookies Baked at Different Temperatures

All the chemical parameters determined on the different cookie samples were influenced by the variable sugar type, time and temperature of baking (Table 1).

Moisture levels decrease with baking time but with some differences between samples. In samples baked for 5 min at 150 °C, the moisture levels were in the range of 18.8 ± 1.7 to 19.7 ± 1.3 g/100 g. No significant differences on moisture levels were found between samples baked at 150 °C up to 15 min, then sucrose cookies lost water faster than the other samples up to 25 min of baking. At the end of the baking process (35 min), the moisture content was similar in all samples. The moisture levels in cookies baked for 5 min at 170 °C were similar, ranging from 17.0 ± 1.5 g/100 g to 17.9 ± 1.3 g/100 g without significant differences between samples. After 10 min of baking up to the end of the baking time, sucrose cookies had the lowest moisture levels while the fructose samples had the highest level. At the highest baking temperature (190 °C), the samples with sucrose have a significantly lower difference moisture content than the other samples, throughout baking time. After 5 min of baking at 150 °C moisture levels were in the range of 14.4 ± 1.2 g/100 g to 17.0 ± 0.8 g/100 g for sucrose and fructose cookies, respectively. Moisture content of sucrose cookies was significant different from the levels determined in the other samples starting from the first sampling. The fructose and glucose cookies had generally a similar moisture level, even if fructose seems to loss water less quickly [36]. This monosaccharide had good humectant properties and it can retain moisture for a long period of time, even at low RH [47]. Moreover, the highest moisture highlighted by the fructose cookies could be reflected in a less crumbly and crunchy product. At the end of baking time no significant differences on moisture levels were found between samples baked at 190 °C. Increasing either the time or temperature of baking caused a decrease in pH and an increase in titratable acidity. The highest pH value and lowest TTA were detected in sucrose cookies at all baking temperatures and time. At 150 °C, pH values after 5 min of baking were approximately 7.2 ± 0.03 – 7.4 ± 0.11, and TTA ranged from 1.7 ± 0.1 to 2.2 ± 0.2 mL NaOH N/10 and no significant differences was observed. At 35 min of baking, fructose and glucose cookies showed a lower significant different pH value (4.4 ± 0.32 and 4.7 ± 0.34, respectively) respect to sucrose cookies (6.5 ± 0.02). At 170 °C, a similar trend of pH and TTA was observed. In samples baked at 190 °C, after 25 min, the pH values ranging from 5.0 ± 0.11 to 3.8 ± 0.12 in sucrose and fructose cookies, respectively and TTA increased up to 10.6 ± 0.3, 20.2 ± 0.2 and 13.2 ± 1.0 mL NaOH N/10 in sucrose, fructose and glucose cookie samples, respectively (Table 1). The deepest reduction in pH and the highest increase in titratable acidity were found in the fructose cookies, due to the degradation reactions of 1,2 and 2,3 enediol species (sugar degradation pathway) rather than via the Maillard reaction [48]. Similar differences due to a lower reactivity of sucrose to the Maillard reaction than monosaccharides were reported [36].

Table 2 summarizes the level of HMF and the DCs in cookie samples as a function of sugar type, temperature and baking time.

Although values are highly variable, at the lowest temperature, 150 °C, HMF was produced after just 5 min of baking. Then, the HMF concentration remained almost constant in all samples up to 25 and 20 min of baking in sucrose cookies and fructose and glucose cookies, respectively. At the end of baking (35 min), the final levels of HMF were 262.2 ± 7.7, 1350.3 ± 3.3 and 451.5 ± 15.7 mg/kg in the sucrose, fructose and glucose cookies, respectively.

At 170 °C, the HMF levels remained constant during the initial stages of baking in all samples. In fructose cookies, the HMF levels increased rapidly after 15 min of baking, reaching the highest HMF level at the end of baking (approximately 639.7 ± 44.1 mg/kg) (Table 2). At the highest temperature, 190 °C, the HMF levels were highly variable; the highest level after 25 min of baking was 10,606.5 ± 104.3, 5626.2 ± 130.6 and 3323.9 ± 81.8 mg/kg in fructose, sucrose and glucose cookies, respectively. As just observed, the use of fructose in the recipe results in the highest levels of HMF; moreover, the control of the baking time should be a strategy to contain the HMF levels as a longer baking time, even by a few minutes, can drastically modify the HMF level in cookies (Table 2).

Levels of HMF determined in this paper were in the range reported for cookies at the estimated optimal baking time (EOBT) [26] and in the range found in a survey of commercial cookies [41,49]. Other studies have highlighted that HMF increases in baked goods after a lag-phase with a reasonable decrease in moisture and a_w_ [30,31,32]. In these studied systems, both titratable acidity and HMF behaved similarly, with an initial lag-phase followed by an exponential increase exhibiting a strong exponential correlation (R2 > 0.9300). Our data suggest that if we considered the EOBT [36] for all the temperatures and types of sugar, it coincides with the end of the lag-phase and the beginning of the exponential increase in HMF.

The use of different sugars in the recipe deeply influenced the formation of DCs (Table 3). At 150 °C, in the sucrose cookies, 3-DG levels increase at 25 min of baking (7.1 ± 1.9 mg/kg) and in the following 5 min its concentration increases tenfold, reaching the level of 508.6 ± 1.5 mg/kg after 35 min of baking. In fructose and glucose cookies, the highest levels of 3-DG were found already after 5 min of baking (approximately 35 mg/kg). Increasing the baking time, the 3-DG concentration increases rapidly, reaching a final level of 3404.5 ± 246.5 and 1731.7 ± 120.8 mg/kg in fructose and glucose cookies, respectively. It is interesting to note that in the glucose cookies, 3-DG quickly reached its highest level (3941.7 ± 169.0 mg/kg) after 25 min, then the level decreased and in the last 5 min of baking, the amount reached 1731.7 ± 120.8 mg/kg. This is the typical behavior of an intermediate: a rapid increase in concentration followed by a decrease [50].

In terms of GO and MGO, their levels in sucrose cookies change from 5 to 35 min of baking from 4.4 ± 0.6 to 11.2 ± 2.4 mg/kg and 9.3 ± 4.6 to 7.9 ± 1.1 mg/kg, respectively. In fructose and glucose cookie samples, GO and MGO increased rapidly during baking time, reaching at the end of baking time 79.9 ± 0.1 and 146.5 ± 0.2 mg/kg, and 102.3 ± 3.0 and 49.3 ± 0.7 mg/kg in fructose and glucose cookies, respectively.

A similar trend for DCs was found during baking at 170 °C. In sucrose cookies, the increase in baking temperature induces an increase in all DCs after 20 min of baking, with a final level of 70.6 ± 9.2, 11.2 ± 0.6 and 14.9 ± 1.7 mg/kg for 3-DG, GO and MGO, respectively. Additionally, at this temperature, cookies with fructose and glucose showed the highest levels of DCs during all baking time. These levels were similar to those determined on the same cookie samples baked at 150 °C with about 5 min more of baking (Table 2). Additionally at this temperature, the lowest values for both GO and MGO were measured in the sucrose cookies, while highest values were measured in the other two type of cookies, particularly in the cookies containing fructose as sweetener.

At 190 °C, in all cookie samples, high levels of the three DCs were rapidly accumulated (Table 2). Analogously to what was observed, at 150 and 170 °C, 3-DG concentration plateaued at approximately 3281.3 ± 45.9 mg/kg when glucose was used in the recipes. This behavior was also observed in fructose cookies: 3-DG increased up to 2144.4 ± 243.9 mg/kg at 20 min of baking and then decreased down to 934.6 ± 82.6 mg/kg. The highest levels of GO and MGO were determined: in sucrose cookies, the levels were approximately 362.6 ± 160.9 mg/kg and 293.0 ± 162.7 mg/kg, similarly to those reported in glucose cookies. In fructose cookies, GO and MGO concentrations reached 3014.2 ± 265.4 mg/kg and 503.0 ± 134.5 mg/kg, respectively. This different trend was probably due to the different formation pathways: GO is formed via sugar oxidation during the heating process, unlike MGO, which is formed by the retroaldolization of the intermediate 3-deoxyglucosulose [51], which is enhanced by the Maillard reaction [40].

The data above clearly demonstrate that substitution of sucrose with either fructose or glucose, particular the latter, deeply modifies the levels of DCs, independent of the baking temperature used. Increasing baking temperature emphasizes these differences.

Moreover, 3-DG were generally highest in glucose cookies, while MGO was highest in the fructose one (Table 2).

### 3.2. The Kinetics of Hazardous Chemical Compounds

First-order kinetics were used to fit the concentrations of HMF, 3-DG, GO and MGO determined in the different cookie samples with time. The linear part of the concentration–time graph was used to determine kinetic parameters; for the worst cases, HMF and MGO at 150 °C, only four points were used. Table 3 reports the kinetic parameters of both HMF and DC formation in cookies.

Concerning HMF, the sucrose cookies showed the following K values: 0.22, 0.32 and 0.46 min^−1^ at the three baking temperatures (Table 3). In this sample, 3.2, 2.2 and 1.5 min were required to double the starting HMF concentration (2A_0_) at 150, 170 and 190 °C, respectively (Table 3). The K values ranged between 0.29 and 0.49 min^−1^ in the cookies with fructose and between 0.25 and 0.41 min^−1^ in those with glucose. The highest Ea value for HMF formation was estimated in the sucrose samples (29.2 kJ/mol). The lowest Ea values were determined in fructose (21.3 kJ/mol) and glucose cookies (20.6 kJ/mol). This result indicates that the baking temperature is more influential on the final levels of HMF than the sugar type.

The complexity of kinetics in Maillard reactions has been very well documented [27]. It arises from the complex reaction network, in which parallel and/or consecutive reactions occur. A single observed rate constant reflects a mixture of elementary rate constants [27]. The rate constants obtained for HMF have similar values, independent of the starting sugar, which suggests that, at these temperatures and in these systems, the inversion of sucrose is not the rate-limiting step. HMF and acrylamide accumulation were modelled in cookies, showing that HMF was derived from the caramelization routes of glucose and/or fructose [35]. A similar value for the HMF rate constant determined at 190 °C in sucrose cookies was reported [35]. Thus, the differences in the final levels of HMF, between sucrose cookies and fructose and glucose ones, could be ascribed to other factors. For instance, it could be related to the amount of residual water in the system. It seems that the caramelization reactions in cookies with sucrose were stopped earlier than the other two cookies. Previous studies have shown that the increase in HMF levels in cookies depends on the activation of dehydration reactions and a reasonable decrease in pH values [29,32]. Moreover, it has been shown that the mobilization of reactants, at the melting point of each sugar, can have a significant impact on reactivity [28]. Data in Table 1 show that cookies with sucrose release water faster than the other two systems, also showing both a lower titratable acidity and higher pH values. These factors confirm a stop in the caramelization reactions in this samples.

The kinetics for 3-DG in sucrose cookie samples exhibited K values of 0.32, 0.33 and 0.50 min^−1^ and 2A_0_ values of 2.2, 2.1 and 1.4 min at 150, 170 and 190 °C, respectively, with an Ea value of 18.4 kJ/mol (Table 3). In fructose and glucose cookies, particularly the latter, the values of K suggest that the formation of 3-DG was less dependent on temperature. Fructose samples had K values of 0.15, 0.17 and 0.21 min^−1^ at 150, 170 and 190 °C, respectively, and its 2A_0_ values ranged between 4.6 and 3.4 min. The glucose system had K values of 0.24 at 150 and 170 °C and 0.27 min^−1^ at 190 °C; its 2A_0_ values ranged between 2.9 and 2.6. These two-model systems exhibited lower values of Ea, 12.1 and 5.0 kJ/mol, respectively. The above data support the proposed mechanistic model and the kinetics of DC formation [18]. The behavior of 3-DG has two distinct phases: 3-DG formation and 3-DG degradation. Furthermore, considering the rate constant for HMF comes from a multistep pathway, similar to 3-DG with formation and degradation routes, the difference between KHMF and K3-DG in glucose cookies at 190 °C was equal to 0.14 min^−1^, which is the same value reported for the reaction: 3-DG→3,4-DG. In addition, a K value of 0.3 min^−1^ for the reaction 3,4-DG→HMF was reported. Thus, our results seem to confirm kinetics determined on a model system constituted by a glucose–flour mixture [28]. Moreover, the use of fructose and glucose in the recipe seems to ensure the optimal conditions for generating this hazardous compound and its levels quickly plateaued. Low values of Ea for 3-DG formation suggest that the rate-limiting step is the diffusion of reactants [52]. The reduction in pH, likely due to the production of carboxylic acids, slows down the Maillard reaction but also indicates the progress of the Maillard reaction [27]. The overall concentration of 3-DG in the glucose cookies seems to be the equilibrium between formation and degradation routes. Degradation routes seem to predominate when the 3-DG concentration reaches 3500–4000 mg/kg, which corresponds to the highest TTA increase and pH decrease, indicating an advanced state of the Maillard reaction and thus a more intense thermal process.

GO formation was significantly influenced by the used sugars (Table 3). In the sucrose cookies at 150 °C, a K value of 0.01 min^−1^ was determined, and this value increased up to 0.21 min^−1^ at 190 °C. The 2A_0_ values ranged between 69.3 min at the lowest temperature and 3.3 min at the highest and the Ea value was 128.5 kJ/mol. Cookies with fructose showed the highest K value at 190 °C (0.36 min^−1^), while those with glucose showed the lowest Ea value (22.5 kJ/mol). Again, the rate constant values for GO, both at 150 and 190 °C, were similar to those already reported, supporting the mechanistic model for GO formation in which glucosone is a precursor of GO [28].

Kinetics for MGO had the lowest K value (0.001 min^−1^) for the sucrose samples at 150 °C and the highest value (0.23 min^−1^) at 190 °C, similar values was found for glucose cookies. The lowest Ea value was determined when fructose was used in the recipe (27.6 kJ/mol), while the highest value (223.4 kJ/mol) was determined in the sucrose cookies. MGO, according to the literature, can originate, by retro-aldol cleavage, both from 1 and 3-deoxyglucosone. A zero-rate constant of MGO formation, from 3-DG was reported at the studied temperatures (160–180–200 °C), while it was very high from 1-DG (2.4 min^−1^, at 200 °C) [28]. Data in Table 3 show that, at least, the reaction 1-DG- > MGO is not the rate-limiting step.

The highest Ea values found for MGO and GO in the sucrose cookies suggest that the systems required very high temperatures to accrue meaningful levels of these molecules.

## 4. Conclusions

For the first time, this paper reported the kinetics of the formation of hazardous chemical compounds such as DCs and HMF in cookies. The replacement of sucrose with fructose or glucose in the cookie recipe results in the highest levels of both HMF and DCs independently from baking temperature and time. If fructose was used as sweetener highest levels of HMF, GO and MGO can be found in cookies, while the use of glucose leads to highest amount of 3-DG. To contain the presence of these compounds in cookie samples, a further strategy seems to be the use of the lowest baking temperature (150 and 170 °C). The baking time depends on the surface color development of cookies and the determined kinetic parameters allows to develop models that predict the formation of HMF and DCs in cookies, enabling modulation of the optimal process conditions. Although the use of glucose or fructose to accelerate the development of the desired surface color in baked goods is a routine practice, the highest level of unhealthy compounds found in this work suggest limiting their use.

## Figures and Tables

**Table 1:** Evaluation of the chemical characteristics of the cookie samples during temperature and time of baking.

Temperature (°C)	Samples	Time (min)	Moisture ^1^	pH	TTA ^2^
150 °C	Sucrose cookies	5	19.1 ± 1.6 a	7.4 ± 0.07 a	1.7 ± 0.1 a
		10	14.2 ± 1.2 a	7.4 ± 0.03 a	1.5 ± 0.1 a
		15	10.8 ± 1.1 a	7.4 ± 0.03 a	1.5 ± 0.1 b
		20	5.4 ± 0.5 b	7.3 ± 0.04 a	1.5 ± 0.1 b
		25	2.2 ± 0.2 b	7.0 ± 0.03 a	1.8 ± 0.2 b
		30	1.1 ± 0.2 b	6.9 ± 0.03 a	2.4 ± 0.1 b
		35	0.4 ± 0.08 a	6.5 ± 0.02 a	3.8 ± 0.2 b
	Fructose cookies	5	18.8 ± 1.7 a	7.2 ± 0.03 a	2.0 ± 0.1 a
		10	16.7 ± 1.1 a	7.3 ± 0.06 a	1.9 ± 0.1 a
		15	14.1 ± 1.3 a	6.9 ± 0.19 b	2.4 ± 0.1 a
		20	9.1 ± 0.9 a	6.9 ± 0.13 b	3.1 ± 0.3 a
		25	6.3 ± 0.4 a	6.5 ± 0.02 b	4.1 ± 0.2 a
		30	3.1 ± 0.7 a	5.6 ± 0.13 b	6.5 ± 0.5 a
		35	0.3 ± 0.05 a	4.4 ± 0.32 b	9.3 ± 0.7 a
	Glucose cookies	5	19.7 ± 1.3 a	7.4 ± 0.11 a	2.2 ± 0.2 a
		10	15.4 ± 1.1 a	7.4 ± 0.08 a	2.0 ± 0.2 a
		15	12.5 ± 0.6 a	7.2 ± 0.17 ab	2.3 ± 0.2 a
		20	7.6 ± 0.3 a	6.9 ± 0.15 b	2.8 ± 0.2 a
		25	5.2 ± 0.6 a	6.6 ± 0.29 b	3.7 ± 0.2 a
		30	1.2 ± 0.5 b	5.9 ± 0.13 b	6.1 ± 0.3 a
		35	0.2 ± 0.04 a	4.7 ± 0.34 b	7.8 ± 0.5 a
170 °C	Sucrose cookies	5	17.0 ± 1.5 a	7.4 ± 0.04 a	1.5 ± 0.1 a
		10	10.6 ± 0.7 b	7.3 ± 0.03 a	1.5 ± 0.1 b
		15	6.3 ± 0.7 b	7.2 ± 0.10 a	1.6 ± 0.1 b
		20	2.1 ± 0.3 c	7.2 ± 0.08 a	1.9 ± 0.2 b
		25	1.0 ± 0.3 c	6.7 ± 0.07 a	3.1 ± 0.4 c
	Fructose cookies	5	17.9 ± 1.3 a	7.4 ± 0.11 a	1.8 ± 0.2 a
		10	13.7 ± 1.1 a	7.1 ± 0.03 b	2.2 ± 0.1 a
		15	9.6 ± 0.9 a	6.9 ± 0.08 a	2.9 ± 0.2 a
		20	6.4 ± 0.4 a	6.3 ± 0.05 b	4.7 ± 0.3 a
		25	3.5 ± 0.2 a	5.3 ± 0.38 b	9.4 ± 0.8 a
	Glucose cookies	5	17.5 ± 0.7 a	7.3 ± 0.04 a	1.9 ± 0.1 a
		10	13.5 ±0.7 a	7.1 ± 0.07 b	2.2 ± 0.2 a
		15	9.8 ± 1.0 a	6.8 ± 0.11 a	2.8 ± 0.2 a
		20	5.2 ± 0.3 b	6.2 ± 0.12 b	3.9 ± 0.2 a
		25	2.8 ± 0.2 b	5.9 ± 0.38 b	6.5 ± 0.3 b
190 °C	Sucrose cookies	5	14.4 ±1.2 b	7.4 ± 0.05 a	1.4 ± 0.1 b
		10	8.6 ± 0.7 b	7.3 ± 0.01 a	1.5 ± 0.1 b
		15	4.5 ± 0.4 b	7.3 ± 0.05 a	1.6 ± 0.1 b
		20	2.4 ± 0.4 c	6.7 ± 0.06 a	2.9 ± 0.2 b
		25	1.6 ± 0.7 a	5.0 ± 0.11 a	10.6 ± 0.3 c
	Fructose cookies	5	17.0 ± 0.8 a	7.2 ± 0.09 a	2.0 ± 0.2 a
		10	11.1 ± 0.8 a	6.9 ± 0.09 b	2.6 ± 0.1 a
		15	6.4 ± 0.2 a	6.2 ± 0.10 b	4.1 ± 0.4 a
		20	4.4 ± 0.3 a	5.3 ± 0.20 b	8.0 ± 0.4 a
		25	1.5 ± 0.3 a	3.8 ± 0.12 b	20.2 ± 0.2 a
	Glucose cookies	5	16.1 ± 1.2 a	7.2 ± 0.05 a	2.2 ± 0.2 a
		10	10.7 ± 0.6 a	7.0 ± 0.09 b	2.7 ± 0.2 a
		15	5.7 ± 0.5 ab	6.5 ± 0.08 b	3.9 ± 0.4 a
		20	3.1 ± 0.2 b	5.8 ± 0.39 b	7.6 ±0.5 a
		25	2.1 ± 0.6 a	4.6 ± 0.12 a	13.2 ± 1.0 b

^1^ g/100 g. ^2^ Total titratable acidity expressed on dry matter as mL NaOH N/10. ^a–c^ Data are presented as the mean ± standard deviation (*n* = 6). Different letters indicate significant differences according to Tukey’s test (*p* ≤ 0.05) among them within temperature and time of baking.

**Table 2:** HMF and DC concentration in cookies as a function of the used sugar in the recipe and baking parameters.

Temperature (°C)	Samples	Time (min)	HMF ^1^	3-DG ^1^	GO ^1^	MGO ^1^
150 °C	Sucrose cookies	5	5.6 ± 2.2 a	nd	nd	9.3 ± 4.6 a
		10	4.2 ± 1.6 a	nd	4.4 ± 0.6 b	7.9 ± 0.4 b
		15	4.8 ± 1.8 a	0.8 ± 0.2 c	9.4 ± 0.2 b	7.8 ± 0.6 b
		20	5.4 ± 2.6 a	4.1 ± 5.4 c	9.72 ± 1.0 a	7.8 ± 1.3 b
		25	5.7 ± 0.8 b	7.1 ± 1.9 c	10.3 ± 0.4 b	8.1 ± 0.5 c
		30	48.1 ± 0.7 b	74.6 ± 4.6 c	10.7 ± 0.4 c	8.0 ± 1.6 c
		35	262.2 ± 7.7 c	508.6 ± 1.5 c	11.2 ± 2.4 c	7.9 ± 1.1 c
	Fructose cookies	5	9.5 ± 3.0 a	34.5 ± 3.4 a	10.5 ± 0.3 a	9.4 ± 0.3 a
		10	7.0 ± 2.5 a	101.1 ± 7.0 ab	10.5 ± 0.6 a	12.7 ± 0.4 a
		15	5.5 ± 2.4 a	241.9 ± 23.8 b	11.0 ± 0.7 a	21.8 ± 0.5 a
		20	5.4 ± 1.8 a	523.9 ± 47.5 b	11.4 ± 0.47 a	30.5 ± 2.8 a
		25	11.8 ± 2.0 a	1045.2 ± 115.0 b	14.1 ± 1.9 b	32.9 ± 1.0 a
		30	152.3 ± 2.3 a	1990. 2 ± 56.5 b	35.0 ± 3.7 b	74.7 ± 3.2 a
		35	1350.3 ± 3.3 a	3404.5 ± 246.5 a	79.9 ± 0.1 b	146.5 ± 0.2 a
	Glucose cookies	5	7.0 ± 1.5 a	35.9 ± 22.4 a	11.1 ± 0.5 a	9.4 ± 1.0 a
		10	4.8 ± 0.5 a	207.5 ± 50.3 a	10.4 ± 0.6 a	8.4 ± 0.7 b
		15	4.2 ± 0.9 a	810.0 ± 160.3 a	11.1 ± 1.1 a	9.3 ± 0.9 b
		20	4.8 ± 0.7 a	2120.9 ± 351.1 a	13.9 ± 2.9 a	11.9 ± 1.0 b
		25	12.6 ±3.0 a	3941.7 ± 169.0 a	26.1 ± 7.7 a	17.9 ± 1.9 b
		30	32.2 ± 5.7 c	3250.5 ± 78.6 a	50.7 ± 4.4 a	30.1 ± 0.7 b
		35	451.5 ± 15.7 b	1731.7 ± 120.8 b	102.3 ± 3.0 a	49.3 ± 0.7 b
170 °C	Sucrose cookies	5	3.7 ± 0.9 a	nd	nd	8.2 ± 0.5 b
		10	5.7 ± 1.7 a	0.5 ± 0.3 c	5.5 ± 0.4 b	7.6 ± 0.4 b
		15	3.8 ± 0.9 a	1.0 ± 0.6 c	9.1 ± 0.4 b	7.51 ± 0.5 c
		20	5.9 ± 1.2 c	7.3 ± 1.5 c	10.5 ± 0.9 a	8.6 ± 0.8 c
		25	93.1 ± 17.1 b	70.6 ± 9.2 c	11.2 ± 0.6 c	14.9 ± 1.7 c
	Fructose cookies	5	4.5 ± 0.9 a	69.2 ± 7.8 a	11.0 ± 0.3 a	11.2 ± 0.7 a
		10	4.1 ± 0.8 a	208.8 ± 20.3 b	10.3 ± 0.7 a	20.8 ± 0.8 a
		15	4.7 ± 0.8 a	481.3 ± 79.9 b	11.1 ± 0.4 a	31.5 ± 1.8 a
		20	76.1 ± 6.1 a	1180.5 ± 174.3 b	16.3 ± 2.9 a	40.1 ± 2.3 a
		25	639.7 ± 44.1 a	2324.0 ± 516.9 ab	105.0 ± 82.8 a	83.0 ± 17.5 a
	Glucose cookies	5	3.7 ± 0.8 a	100.4 ± 42.7 a	10.8 ± 0.7 a	8.7 ± 0.5 b
		10	3.1 ± 0.1 a	591.5 ± 32.6 a	10.0 ± 0.6 a	9.6 ± 0.9 b
		15	3.7 ± 0.2 a	1846.2 ± 120.3 a	11.4 ± 1.2 a	12.5 ± 0.7 b
		20	15.3 ± 2.1 b	3490.9 ± 213.1 a	15.3 ± 2.0 a	18.1 ± 1.6 b
		25	170.5 ± 34.9 b	3204.5 ± 348.1 a	36.9 ± 15.2 ab	47.1 ± 1.7 ab
190 °C	Sucrose cookies	5	10.7 ± 7.1 a	nd	nd	7.8 ± 0.4 b
		10	6.9 ± 3.9 a	1.5 ± 0.5 c	8.7 ± 0.9 a	7.2 ± 0.5 b
		15	8.7 ± 2.1 b	5.8 ± 1.0 c	10.9 ± 1.4 a	9.10 ± 0.6 c
		20	97.5 ± 10.7 b	77.4 ± 5.9 b	11.6 ± 0.4 b	14.7 ± 1.6 b
		25	5626.2 ± 130.6 b	1197.2 ± 27.7 b	362.6 ± 160.9 b	293.0 ± 162.7 ab
	Fructose cookies	5	15.6 ± 7.6 a	100.3 ± 7.1 b	9.0 ± 0.4 a	13.2 ± 0.8 a
		10	8.7 ± 2.0 a	331.2 ± 41.9 b	11.5 ± 2.1 a	28.9 ± 2.6 a
		15	22.1 ± 6.3 ab	963.9 ± 101.1 b	14.1 ± 1.3 a	38.1 ± 1.1 a
		20	444.7 ± 107.8 a	2144.4 ± 243.9 a	45.5 ± 5.8 a	118.2 ± 22.1 a
		25	10606.5 ± 104.3 a	934.6 ± 82.6 b	3014.2 ± 265.4 a	503.0 ± 134.5 a
	Glucose cookies	5	16.4 ± 7.6 a	211.6 ± 17.1 a	10.0 ± 0.7 a	8.8 ± 0.7 b
		10	8.9 ± 4.3 a	1251.4 ± 111.5 a	10.8 ± 1.10 a	12.1 ± 2.0 b
		15	27.5 ± 2.9 a	3281.3 ± 45.9 a	15.2 ± 3.8 a	19.1 ± 0.4 b
		20	310.5 ± 193.2 a	2913.6 ± 659.7 a	46.7 ± 15.4 a	105.3 ± 30.4 a
		25	3323.9 ± 81.8 c	2060.0 ± 81.2 a	249.1 ± 126.1 b	314.1 ± 72.7 ab

^1^ mg/kg dry matter. Data are presented as the mean ± standard deviation (*n* = 6). ^a–c^ Different letters indicate significant differences according to Tukey’s test (*p* ≤ 0.05) among theses within temperature and time of baking.

**Table 3:** Kinetic parameters for HMF and DC formation in cookies.

Sucrose Cookies				Fructose Cookies				Glucose Cookies			
T	K	2A_0_	Ea ^1^	T	K	2A_0_	Ea ^1^	T	K	2A_0_	Ea ^1^
(°C)	min^−1^ (R^2^)	min		(°C)	min^−1^ (R^2^)	min		(°C)	min^−1^ (R^2^)	min	
	HMF		29.2 (7.0)		HMF		21.3 (5.1)		HMF		20.6 (4.9)
150 °C	0.22 (0.85)	3.2		150 °C	0.29 (0.85)	2.4		150 °C	0.25 (0.91)	2.8	
170 °C	0.32 (0.82)	2.2		170 °C	0.34 (0.91)	2.0		170 °C	0.38 (0.98)	1.8	
190 °C	0.46 (0.88)	1.5		190 °C	0.49 (0.95)	1.4		190 °C	0.41 (0.97)	1.7	
	3-DG				3-DG				3-DG		
150 °C	0.32 (0.95)	2.2	18.4 (4.4)	150 °C	0.15 (0.99)	4.6	12.1 (2.9)	150 °C	0.24 (0.96)	2.9	5.0 (1.2)
170 °C	0.33 (0.95)	2.1		170 °C	0.17 (0.99)	4.0		170 °C	0.24 (0.95)	2.9	
190 °C	0.50 (0.99)	1.4		190 °C	0.21 (0.99)	3.4		190 °C	0.27 (0.97)	2.6	
	GO				GO				GO		
150 °C	0.01 (0.86)	69.3	128.5 (30.7)	150 °C	0.08 (0.77)	8.7	61.2 (14.6)	150 °C	0.12 (0.96)	6.0	22.5 (5.4)
170 °C	0.05 (0.84)	15.4		170 °C	0.13 (0.77)	5.3		170 °C	0.08 (0.86)	8.7	
190 °C	0.21 (0.67)	3.3		190 °C	0.36 (0.79)	1.9		190 °C	0.20 (0.93)	3.5	
	MGO				MGO				MGO		
150 °C	0.001 (0.90)	693.0	223.4 (53.4)	150 °C	0.09 (0.95)	8.0	27.6 (6.6)	150 °C	0.09 (0.98)	8.2	39.2 (9.4)
170 °C	0.16 (0.82)	16.5		170 °C	0.10 (0.95)	6.3		170 °C	0.10 (0.89)	7.0	
190 °C	0.23 (0.76)	3.1		190 °C	0.17 (0.94)	4.1		190 °C	0.22 (0.96)	3.0	

^1^ Ea expressed as kJ/mol, and as kcal/mol in parenthesis.

## Data Availability

All data supporting this study’s findings are included in this article.
